# Theory of Bowstring Disease: Diagnosis and Treatment Bowstring Disease

**DOI:** 10.1111/os.12417

**Published:** 2019-03-04

**Authors:** Jian‐gang Shi, Xi‐ming Xu, Jing‐chuan Sun, Yuan Wang, Qing‐jie Kong, Guo‐dong Shi

**Affiliations:** ^1^ Department of Orthopedic Surgery, Spine Center Changzheng Hospital, Second Military Medical University

**Keywords:** Bowstring disease, Disc degeneration, Low back pain, Nerve stretch, Scoliosis

## Abstract

Bowstring disease (BSD) is a new classification of spine disease caused by axial stretched lesion on nerve roots and the spinal cord, which is differentiated from disc herniation and canal stenosis in that it is caused by nerve compression lesions. BSD could be caused by mismatched growth rates between the spine and nerve roots (the juvenile type), or by imbalanced degenerative rates between the spine column and nerve roots (degenerative type). Here, we propose that there are several self‐adjust mechanisms to relieve axial nerve tension: (i) nerve growth; (ii) posture adjustment and low back pain; (iii) autogenous degeneration of intervertebral disc; and (iv) idiopathic and degenerative scoliosis. Iatrogenic lesions could also result in BSD, which could be presented as adjacent segment degeneration, leading to adding‐on effects and other neurological symptoms. The diagnosis criteria are proposed based on symptoms, physical examination, and radiological presentations. To remove axial tension on nerve roots, lumbar surgery should aim to restore the coordination of spine and cord units. Capsule surgery, shortening the spine column, could decompress cord and nerve roots 3‐dimensionally.

Since the invention of laminectomy to treat lumbar disc herniation by Dr Smith in 1829, spine surgery has been evolving toward minimal invasiveness and greater accuracy. Specifically, with the advent of the posterior pedicle screw system, spinal deformity, neoplasm, degeneration, and trauma can be treated effectively. However, some spine surgeons come to notice the dilemma despite modern techniques and advanced theories: (i) many patients complain of back or leg pain without obvious nerve compression (these individuals are commonly left undiagnosed or untreated); and (ii) some patients suffer neurological deterioration or even foot‐drop and tethered cord syndrome (TCS) after routine back surgery (typically, there are no obvious postoperative radiographic findings). These issues are quite common and challenging for spine surgeons. To address these concerns, Dr Shi and his team proposed the theory of bowstring disease (BSD)^1^. The theory of BSD addresses common concerns regarding nerve root and spinal cord axial stretch lesions, which could revolutionize surgery philosophy.

## Definition of Bowstring Disease

Bowstring disease (BSD) is defined as axial hypertension on nerve roots and the spinal cord caused by congenital anomalies, degeneration or other issues, which may be accompanied by other lesions, such as lumbar disc herniation, spinal cord stenosis or spondylolisthesis, or be aggravated by iatrogenic lesions, resulting in neurological symptoms. Worse symptoms indicate higher axial tension on nerve roots and dura, as found during surgery. The shape of the lumbosacral spine resembles a bow, and the stretched nerve roots and dura resemble a string, thus comes the name “bowstring disease.”

## Incidence of Bowstring Disease

Neurosurgeons have generally not paid attention to nerve root and cord axial stretch lesions, and the incidence of BSD has not been verified in large‐scale epidemiological studies. Yet, based on the present authors’ experience, at least 10% of outpatients in spine departments are suspected to have BSD. Patients are generally presented as a three‐peak distribution: (i) adolescent children, during their adolescent growth peak or 1 to 2 years after the peak, mostly with low back pain as the main complaint; (ii) adults in their early 30s with aggravating symptoms, who are often neglected in outpatient clinics and have a long history of suffering; and (iii) elderly patients with lumbar degenerative diseases (e.g. lumbar disc herniation or lumbar canal stenosis, etc.), who have high risk of postoperative neurological complications after routine surgeries, such as foot‐drop or contralateral leg pain. Still, large‐scale clinical investigation is required from neurosurgeons worldwide.

## Etiology of Bowstring Disease

The pathological basis of lumbosacral nerve BSD is a nerve axial traction injury. Studies have found that the spinal cord and nerve roots exhibit a kind of elasticity like rubber^2^, ^3^, ^4^. Singh *et al.* found that nerve roots could be elongated with decreased cross‐sectional area, when pulled statically or dynamically^2^. Electrophysiological monitoring revealed that as the nerve root pulling force increased, nerve conduction velocity and potential amplitude gradually decreased, the integrated action potential area became smaller or even disappeared, and, finally, nerve root conduction was completely blocked^3^. Stretching tension can not only directly lead to abnormal nerve conduction, but can also reduce the blood flow of nerve roots and cause nerve ischemia damage. Kobayashi *et al.* found that when radial pain occurred during a straight leg raising test, the nerve root blood flow of both L_5_ and S_1_ decreased by more than 70%^4^. Takamori *et al.* performed a straight leg raising test during surgery and found that the S_1_ innervated muscle‐evoked potential and blood supply were significantly reduced^5^.

Nerve roots are prone to mechanical lesions and their resistance to pulling is merely 10% of the peripheral nerves, to a large extent, due to a thin outer membrane^6^, ^7^. In particular, dorsal root ganglion cells are not only sensitive to various inflammatory factors, ischemia, and hypoxia, but are also more susceptible to mechanical injury^8^, ^9^, ^10^. Nerve roots penetrate from the dural sac, transverse to the lateral recess, and pop out the nerve root foramen. Nerve roots can be divided into three segments: the disc part, the pedicle part, and the foramen part^11^. The disc part is the initial segment with the dural sac transforming to a sheath around the nerve root. This segment is vulnerable to compression and traction due to disc herniation. The canal part has radial ligaments anchoring the nerve roots to the canal wall. Under physiological conditions, these ligaments can prevent external stress from being transmitted to the nerve roots in the spinal canal^12^, ^13^. However, when nerve roots are stretched inside the canal, the ligaments can also prevent strain from spreading out to the peripheral nerves. Thus, the dural sac part and the foramen anchoring part together are the anatomical basis for tension on nerve roots.

**Figure 1:**
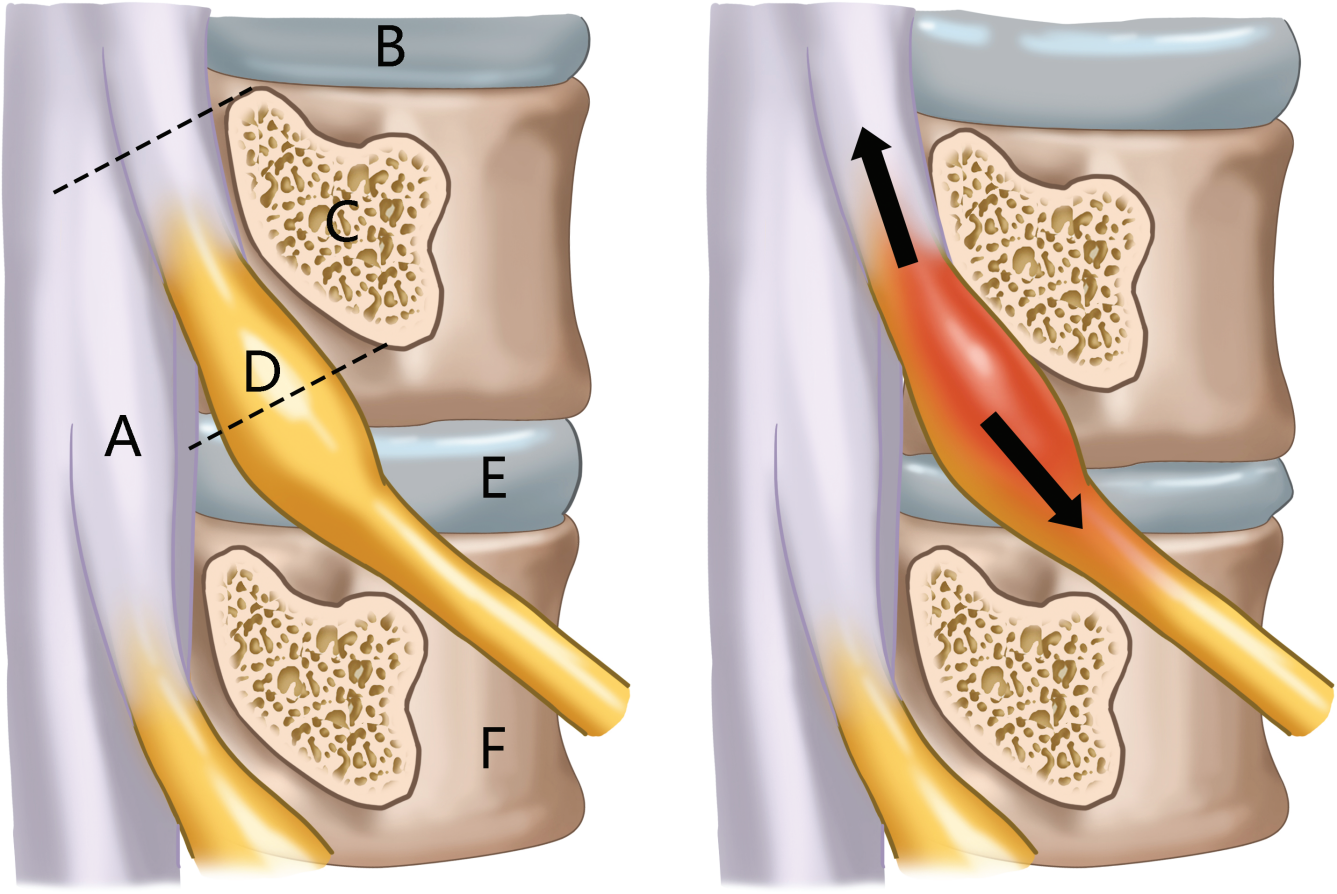
Diagram of bowstring disease. (A) Normal nerve root. (B) Developmental bowstring disease (BSD) due to quicker growth of vertebra than nerve root, resulting in tension on the nerve root. (C) Degenerative BSD: as the nerve root degenerates faster than the disc with less elasticity, tension on the nerve root results.

How does stretching tension develop on nerve roots? We proposed two different pathogenic mechanisms of nerve root stretch injuries: a juvenile type due to spine and nerve root development mismatch; and a degenerative type due to mismatched degenerative rates of spine and nerve roots (Fig. 1). When height spurts during puberty, nerve root axial tension can appear if nerve roots grow slower than the vertebral column. However, physiological tension can promote nerve growth when the tension is beyond the normal range; this can destroy the anatomical structure and physiological function of nerve roots^14^, ^15^, ^16^, ^17^. This process is referred to as a spine and nerve developmental mismatch (Fig. 1). Most adolescent BSD cases are due to this reason. Some patients experience height increases of 20 cm per year.

Why does BSD also present in middle‐aged and elderly patients with spine degeneration and loss of intervertebral space? We believe mismatch between spine and nerve still exists not due to growth but to degeneration. Nerve roots degenerate faster than the spinal column, resulting in passive tension on nerve roots. In elderly patients, nerve roots become less elastic, as do blood vessels, which makes nerve roots more susceptible to stretch injury (Fig. 1). Therefore, there is high risk of iatrogenic nerve root stretch during conventional lumbar surgery when the axial status of nerve roots is ignored, especially when restoring lumbar spondylolisthesis or correcting scoliosis (Fig. 1).

## Diagnostic Criteria of Bowstring Disease

A preliminary diagnostic criterion has been established according to clinical findings.

### *Clinical Presentations*

Patients usually complain of persistent back pain (lasting for years), which can be aggravated when lying down or bending, or in the morning. Leg pain and numbness might be bilateral or lateral, which could be partly relieved by rest. Severe cases may present with numbness of the perineum, and dysfunction of urination and defecation.

### *Physical Examination*

Usually, bilateral knee tendon reflexes are active or hyperactive. If there is severe lumbar disc herniation or lumbar spinal stenosis, knee reflex may be weakened or absent. Unilateral or bilateral straight leg raising or Fehling tests would be positive. The anal sphincter muscle strength might decrease in severe cases.

### *Imaging Examination*

There would be no sign of nerve root compression on lumbar MRI images (excluding cervical and thoracic lesions). The cauda equina sedimentary sign would be positive. Future functional MRI may provide new evidence.

### *Electromyogram Examination of Lower Limbs*

An electromyogram would show neurogenic lesions in bilateral or unilateral lower extremities: small MCV and SCV, extended latency, and amplitude less than 50% performance.

### *Intraoperative Findings*

The dural sac and nerve roots would be stiff. There would be resistance to touching nerve roots with a probe. The motion of the nerve root would be weakened or diminished.

## Compensatory Mechanism of Bowstring Disease

As BSD is a disease related to growth or degeneration, there are several compensatory mechanisms that will restore the alignment of the spinal column and nerve roots (or cord). According to the author’s clinical experience and related research reports, the self‐adjust mechanisms are as follows^14^, ^18^.

### *Nerve Growth*

Although overstretching can destroy nerve structures, studies have found that neurons can adapt to low and moderate tension^14^, ^15^, ^16^. It is reported that nerves are under tension during growth. When the joints are in motion, the nerves can also withstand high tension exceeding the initial value of 25%. During fracture healing and long bone lengthening, the nerves can adapt to certain tension^19^. Cytological experiments have found that moderate tension can accelerate neuronal regeneration and axonal growth^20^, ^21^. in vivo animal studies have found that elongation of 11% of the sciatic nerve not only does not destroy neural signals but also promotes neural and myelin regeneration and synthesis of structural proteins^22^. The response of nerves and myelin to tension is gaining increasing attention by researchers.

### *Posture Adjustment and Low Back Pain*

When axial nerve root tension exceeds tolerance, the body adjusts the posture to reduce the tension. Contraction of back muscles will increase the lordosis, reduce the height of the posterior column, and reduce the route of nerve roots in the lateral recess, and, thereby, relieve the stretch. The increase of lumbar lordosis in patients results in greater pressure at the articular joints, followed by articular process hyperplasia, joint capsule damage, osteoarthritis, and other chronic inflammatory lesions, which cause low back pain^18^, ^23^. Therefore, lower back pain in young patients is mostly caused by increased lumbar facet pressure; lower back pain in elderly patients is mostly due to chronic inflammatory lesions of articular joint. Although there are many hypotheses to explain the pathogenesis of low back pain, such as discogenic pain, muscle strain, and aseptic inflammation, it remains elusive^24^. Based on bowstring theory, the author believes that low back pain is an accompanying consequence of posture adjustment to relieve nerve root tension.

### *Autogenous Degeneration of Intervertebral Discs*

**Figure 2:**
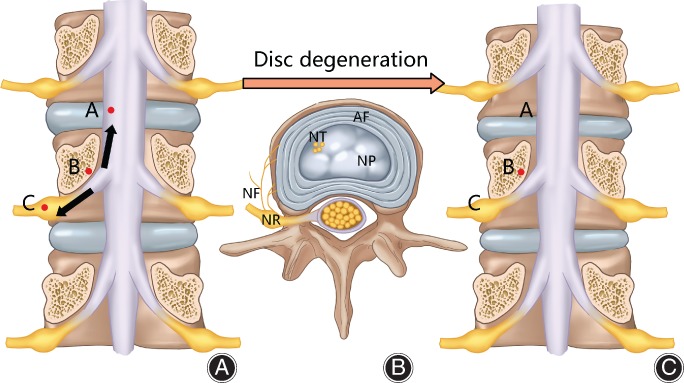
Autogenous disc degeneration. (A) Axial tension on nerve root. (B) Initiation of disc degeneration. Usually there are no nerve fibers in the nucleus pulposus, while high traction of nerve roots initiates disc degeneration through a kind of neural reflex arc. Innervation and neurotransmitters could be found in degenerated nucleus pulposus. (C) Autogenous disc degeneration could relieve the tension on the nerve root. AF, annulus fibrosus; NF, nerve fiber; NP, nucleus pulposus; NR, nerve root; NT, neurotransmitter.

The spinal column includes vertebrae and intervertebral discs. The height of the spine could change as discs are compressed or regain their height, making discs an essential buffer to coordinate the spine and nerve roots. When nerve roots are stretched, a disc could degenerate, reducing its height and, thus, shortening the course of nerve roots and relieving the tension on nerves (Fig. 2). Therefore, we could easily find disc height loss in the elderly without any disc herniation. The disc could be “eaten” by macrophages, with reduction in the extracellular matrix of the nucleus, and, thus, loss its height. Patients with BSD often show multiple disc height loss, as evidenced by MRI T_2_ images, without obvious disc herniation or prolapse, especially in young patients. Usually, the degenerated discs are not the ones that bear most gravity, like L_4‐5_ or L_5_S_1_. If the disc cannot degenerate autogenously, disc herniation might occur. Protrusion or prolapse of the nucleus will reduce the disc height and abolish the tension on nerve roots. Higher tension might be found in the disc herniation segment than in degenerative discs. Therefore, there is risk of iatrogenic nerve root stretch when restoring disc height during lumbar surgery Foot‐drop and ipsilateral neurological lesions can result after lumbar spondylolisthesis surgery, deformity correction surgery, or micro‐invasive surgery. However, how nerve root tension initiates disc degeneration lacks research. The reflex between nerve roots and disc degeneration may provide new vision for future research.

### *Idiopathic Scoliosis*

During growth, a variety of nerve root or connective tissue abnormalities might occur. If the abnormalities are asymmetrical, the axial tension would not be even on bilateral nerve roots. To re‐balance the tension on nerve roots, the spine column could rotate or bend the canal, and, thus, result in scoliosis. The scoliosis frequently progresses with spine growth. According to the author’s experience, nerve root tension is higher on the concave side than on the convex side, which requires nerve root and connective tissue release. However, if the scoliosis progresses progressively, decompensation may occur; that is, nerve roots on the convex side may also have stretch tension. Therefore, idiopathic scoliosis may be an auto‐adjustment due to BSD. Anatomical studies have found that in patients with scoliosis, the length of the anterior column of the spine is much larger than the length of the spinal canal and posterior column, and the degree of rotation of the vertebral body is positively correlated with the reduction of the length of the canal^25^. MRI studies have also found the ratio of the length of the spinal canal to the vertebral body to be significantly lower than that of a normal control group^26^. Therefore, we believe that the vertebral rotation and scoliosis is an essential self‐repair approach to restore the balanced relationship of spine and nerve roots during growth.

### *Degenerative Scoliosis*

Degenerative scoliosis is a similar condition occurring during aging. The nerve root degenerates faster (less nerve root elasticity) than the spine column, which causes nerve root stretch. If the tension were imbalanced on bilateral sides, vertebral rotation would appear. In addition, the disc wedges relieve the tension on the concave side of the nerve root, helping to form scoliosis. Considering the fragility of nerve roots in elderly patients, the compensation of scoliosis might not be enough to remove the stretch, in which case neurological symptoms appear. Many clinical studies have used conventional methods to treat degenerative scoliosis^27^, ^28^. After spine surgery, serious neurological complications such as foot‐drop and cauda equina syndrome might, to some extent, occur, yet no obvious nerve damage has been found by MRI investigation in these cases. We speculate that nerve roots might be stretched when restoring the intervertebral height of the concave side during correction of scoliosis. Therefore, according to our clinical experiences and the theory of BSD, the principle to treat degenerative scoliosis is to restore the balance of the spine and nerve roots. Scoliosis is caused by abnormal tension on unilateral nerve roots. The lordosis or kyphosis is caused by abnormal tension on bilateral nerve roots, and it is necessary to resolve bilateral nerve root tension.

## Iatrogenic Bowstring Disease

### *Adjacent Segmental Degeneration*

When a large cage is inserted to restore intervertebral height, the radiating ligaments anchoring nerve roots at the foramen can cause high nerve root tension. The disc is relatively soft compared with vertebra and can change height. Thus, the upper level disc might degenerate to decrease its height and relieve the nerve root tension. This could explain why adjacent segmental degeneration (ASD) frequently affects the proximal segment. Because the nerve roots from the distal proximal segment are located below the disc, the nerve root tension on the lower adjacent segment is less affected. At present, there is considerable debate on the cause of ASD. Some scholars believe that neighboring spondylosis is a natural degenerative course^29^. Others think internal fixation is the essential cause^30^. Long‐term follow‐up demonstrates that ASD is correlated with follow‐up time but not with the patient’s age, fusion segment, and sagittal sequence^31^. We regard ASD to be a self‐repair approach that alleviates axial tension on lower nerve roots^28^.

### *Adding‐on Effect*

Similar to the mechanism of ASD, if the axial tension on the nerve root is not relieved during surgical correction of scoliosis, especially after distraction of the concave side, scoliosis will progress to relax the tension on the nerve root and dural sac. Severe complications of scoliosis surgery include postoperative pain, numbness of lower extremities, foot drop, and even cauda equina syndrome. The adding‐on effect is defined as newly onset of curve or the adjascent disc wedging more than 5° distal to the internal fixation, which is consistent with the main curve with minimal follow up of 2 years^31^. The reported incidence is as high as 21%. This phenomenon will gradually reverse the correction of scoliosis. Revision surgery might be needed in severe cases. It is reported in the literature that this phenomenon might be related to improper selection of the lower fixed vertebra, curve type, and shoulder balance. We believe the adding‐on effect is due, to a large extent, to unrelieved axial tension on nerve roots^32^, ^33^, ^34^.

### *Iatrogenic Bowstring Disease*

Iatrogenic BSD might, if fact, be common as many surgeons do not take axial tension into account. For patients with low back pain, the incidence of aggravated symptoms after traction is 7% to 31%, and some patients even need surgery after traction^35^, ^36^. After routine lumbar surgery, the incidence of neurological complications was 2% to 8%, and most patients did not reveal nerve compression after MR examination^37^, ^38^. The New York Hospital for Special Surgery reported that 7 in 244 patients undergoing lateral interbody fusion had contralateral lower extremity dyskinesia. The authors speculated that the expansion of intervertebral space might be the culprit^39^.

Spine deformity surgery is also an important cause of lumbosacral nerve BSD. According to the Scoliosis Research Association, the rate of new nerve injury after scoliosis in children is 0.4% to 2.0%^40^, ^41^, ^42^, ^43^. The incidence of new neurological complications after adult scoliosis is 1.8% to 3.1%, and some patients present with equine syndrome. However, no compression or nerve injury was found on routine imaging studies^27^, ^28^.

## Surgical Strategy

**Figure 3:**
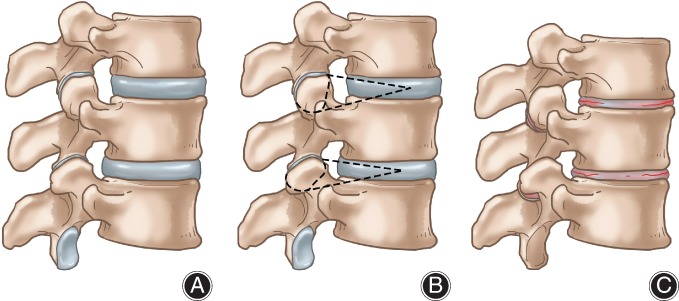
Capsule surgery. (A) Normal lumbar spine. (B) Facet joint and disc are going to be dissected; the interspinous ligament could be also dissected. (C) Effects after capsule surgery: the intervertebral space is shortened, lordosis is increased, and the spine column is shortened evenly.

At present, the principle of conventional lumbar surgery is to relieve nerve root compression and restore spine alignment, while ignoring whether the nerve root is stretched or not. Most surgeons do not pay attention to the degeneration of the nerve root and axial tension. Based on the anatomy of the spinal column and nerve roots, our institute proposes a capsule surgery: a nerve root axial decompression technique to restore the coordination between column and cord (Fig. 3)^1^.

The purpose of spinal surgery is not only to restore the normal sequence, but, more importantly, to decompress nerve roots 3‐dimensionally, to restore the coordination of the spine and cord functional units. Capsule surgery can relieve nerve root hypertonicity in two ways: on the one hand, by removing 2/3 of the posterior intervertebral discs and interspinous ligaments, compressing the posterior column, and reducing the course of nerve roots; and, on the other hand, by partially removing the upper and lower facets of the foramen, partly destroying the ligaments anchoring nerve roots. Capsule surgery could relieve nerve root compression and axial tension at the same time. The key is to restore the natural state of nerve roots. This surgical approach is particularly suitable for developmental disorders such as scoliosis and TCS, to prevent disease progression.

## Tethered Cord Syndrome

Tethered cord syndrome is defined as tethered cord with conus below L_2_ due to spina bifida or other congenital abnormalities^44^. Patients present with a wide variety of signs and symptoms, including cutaneous stigmata, skeletal deformity, motor defects, and sphincter dysfunction^45^. Usually the neurological lesions deteriorate with age. The current surgical strategy is the untethering technique, dissecting the filum terminale and resecting other connective tissues (lipoma) to release the cord^46^. Yet, recent aggregated evidence fails to support the benefits of surgery over conservative therapy^47^. We performed a long‐term follow‐up (13.7 years on average) of 109 patients with meningomyelocele who underwent untethering surgery. Results showed limited improvement of bladder function and high risk of deterioration. We interviewed over 600 TCS patients from seven cities of mainland China and found that bladder dysfunction appeared at 4.9 years of age and deteriorated at 11.8 years of age. Onset and aggravation of neurological lesions was closely correlated with growth peak. Considering the close relationship between lumbar spine and cord during growth, we propose that TCS is due to abnormal cord stretch caused by column growth. In TCS patients, the cord has been attached and loses its ability to go upward freely during growth. When the lumbar column grows fast during puberty, the cord is inevitably being stretched by the mismatch between the cord and column height. Therefore, TCS is a medullary‐specific BSD. Multiple segments capsule surgery has been found to have excellent outcomes for bladder function in TCS patients, and could alleviate the cord traction and prevent further stretch during growth.

## Summary

**Figure 4:**
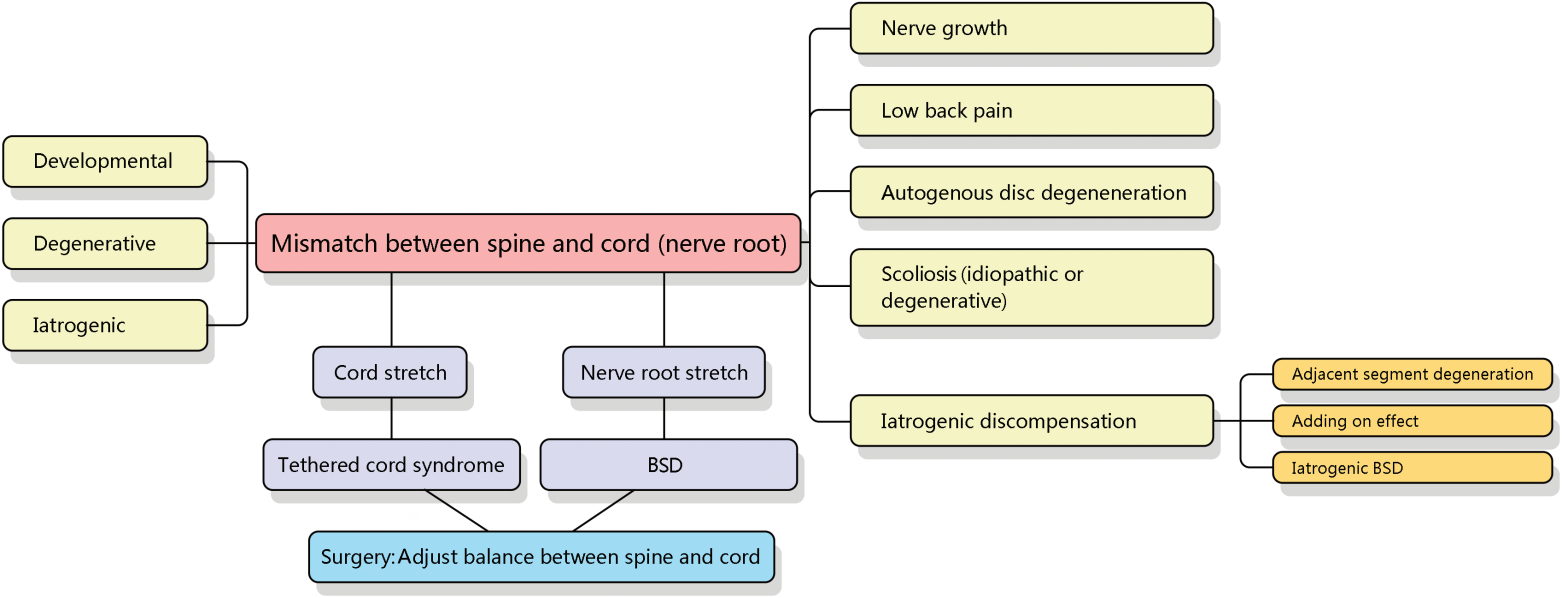
Theoretical framework of bowstring disease (BSD). BSD is caused by axial tension on the nerve root, which results from developmental, degenerative, and iatrogenic lesions. Cord stretch leads to tethered cord syndrome, while nerve root stretch results in BSD. Both conditions could be treated by capsule surgery to re‐balance the relation between spine and cord. When the nerve root is stretched, the body has the following ways to compensate: accelerating nerve growth; adjustment of posture (low back pain would appear due to persistent pressure on the facet joint and muscle contraction); autogenous disc degeneration to reduce the intervertebral space and relieve the traction on nerve root; and idiopathic or degenerative scoliosis would occur due to disc wedging and vertebra rotation. BSD theory could also explain the mechanism of adjacent segment degeneration, the adding‐on effect, and iatrogenic neurological deteriorations.

In the theoretical frame of BSD, the pathogenesis of low back pain, intervertebral disc degeneration, spinal deformity, and adjacent segment degeneration can be explained (Fig. 4). In addition, the surgical plan can be guided to prevent scoliosis progress and iatrogenic complications. At present, the study of BSD is still in its infancy. In particular, more specific radiologic or laboratory diagnostic indices are needed to determine the degree and extent of neural injury. Further joint research is warranted.
